# Artificial Intelligence-Enabled Electrocardiogram Predicted Left Ventricle Diameter as an Independent Risk Factor of Long-Term Cardiovascular Outcome in Patients With Normal Ejection Fraction

**DOI:** 10.3389/fmed.2022.870523

**Published:** 2022-04-11

**Authors:** Hung-Yi Chen, Chin-Sheng Lin, Wen-Hui Fang, Chia-Cheng Lee, Ching-Liang Ho, Chih-Hung Wang, Chin Lin

**Affiliations:** ^1^Department of Internal Medicine, National Defense Medical Center, Tri-Service General Hospital, Taipei, Taiwan; ^2^Division of Cardiology, Department of Internal Medicine, National Defense Medical Center, Tri-Service General Hospital, Taipei, Taiwan; ^3^Department of Family and Community Medicine, National Defense Medical Center, Tri-Service General Hospital, Taipei, Taiwan; ^4^Department of Internal Medicine, National Defense Medical Center, Tri-Service General Hospital, Taipei, Taiwan; ^5^Artificial Intelligence of Things Center, National Defense Medical Center, Tri-Service General Hospital, Taipei, Taiwan; ^6^Medical Informatics Office, National Defense Medical Center, Tri-Service General Hospital, Taipei, Taiwan; ^7^Division of Colorectal Surgery, Department of Surgery, National Defense Medical Center, Tri-Service General Hospital, Taipei, Taiwan; ^8^Division of Hematology and Oncology, Department of Internal Medicine, National Defense Medical Center, Tri-Service General Hospital, Taipei, Taiwan; ^9^Department of Otolaryngology-Head and Neck Surgery, National Defense Medical Center, Tri-Service General Hospital, Taipei, Taiwan; ^10^National Defense Medical Center, Graduate Institute of Medical Sciences, Taipei, Taiwan; ^11^Medical Technology Education Center, National Defense Medical Center, School of Medicine, Taipei, Taiwan

**Keywords:** artificial intelligence, electrocardiogram, deep learning, heart failure, ejection fraction, left ventricular end-diastolic diameter, cardiovascular outcome

## Abstract

**Background:**

Heart failure (HF) is a global disease with increasing prevalence in an aging society. However, the survival rate is poor despite the patient receiving standard treatment. Early identification of patients with a high risk of HF is important but challenging. Left ventricular end-diastolic diameter (LV-D) increase was an independent risk factor of HF and adverse cardiovascular (CV) outcomes. In this study, we aimed to develop an artificial intelligence (AI) enabled electrocardiogram (ECG) system to detect LV-D increase early.

**Objective:**

We developed a deep learning model (DLM) to predict left ventricular end-diastolic and end-systolic diameter (LV-D and LV-S) with internal and external validations and investigated the relationship between ECG-LV-D and echocardiographic LV-D and explored the contributions of ECG-LV-D on future CV outcomes.

**Methods:**

Electrocardiograms and corresponding echocardiography data within 7 days were collected and paired for DLM training with 99,692 ECGs in the development set and 20,197 ECGs in the tuning set. The other 7,551 and 11,644 ECGs were collected from two different hospitals to validate the DLM performance in internal and external validation sets. We analyzed the association and prediction ability of ECG-LVD for CV outcomes, including left ventricular (LV) dysfunction, CV mortality, acute myocardial infarction (AMI), and coronary artery disease (CAD).

**Results:**

The mean absolute errors (MAE) of ECG-LV-D were 5.25/5.29, and the area under the receiver operating characteristic (ROC) curves (AUCs) were 0.8297/0.8072 and 0.9295/0.9148 for the detection of mild (56 ≦ LV-D < 65 mm) and severe (LV-D ≧ 65 mm) LV-D dilation in internal/external validation sets, respectively. Patients with normal ejection fraction (EF) who were identified as high ECHO-LV-D had the higher hazard ratios (*HR*s) of developing new onset LV dysfunction [*HR*: 2.34, 95% conference interval (*CI*): 1.78–3.08], CV mortality (*HR* 2.30, 95% *CI* 1.05–5.05), new-onset AMI (*HR* 2.12, 95% *CI* 1.36–3.29), and CAD (*HR* 1.59, 95% *CI* 1.26–2.00) in the internal validation set. In addition, the ECG-LV-D presents a 1.88-fold risk (95% *CI* 1.47–2.39) on new-onset LV dysfunction in the external validation set.

**Conclusion:**

The ECG-LV-D not only identifies high-risk patients with normal EF but also serves as an independent risk factor of long-term CV outcomes.

## Introduction

Heart failure (HF) is a common clinical entity with increasing prevalence in an aging society, which affects 5.7 million patients and more than 870,000 new cases are diagnosed in the United States every year (1). In developed countries, about 2% of the population lives with HF (1, 2). The American Heart Association forecasted that total costs associated with HF were at $20.9 billion in 2012 and are projected to rise to $53.1 billion by 2030 (3). Currently, HF is classified as reduced ejection fraction (HFrEF), mildly reduced ejection fraction (HFmrEF), and preserved ejection fraction (HFpEF) based on different ejection fraction (EF) levels (4). Multiple modality treatment for the patients with HF, such as renin-angiotensin system inhibition, beta-blocker, and aldosterone antagonist, is evidence-based and recommended in guidelines (4, 5). However, even with treatment, the HF survival rate remains poor globally and the mortality ranged from 17 to 45% in a year among the patients who were admitted to a hospital because of HF (1, 2, 6). Such evidence points out the significant problem of HF in aged society. Early identification of those patients who are at risk to develop HF and adequate risk reduction helps to improve the quality of life, reduce hospitalization, and promote survival outcomes.

In patients with HF, there were several important parameters for the assessment of cardiac functional and structural changes. As EF was the ratio of blood leaving heart each time it contracts, the left ventricular end-diastolic diameter (LV-D) and end-systolic diameter (LV-S) influenced the value of ECHO-EF. The principal ECG changes in patients with increased LV-D and LV-S in LV hypertrophy include augmented QRS amplitude, prolonged QRS conduction time, changes in instantaneous and mean QRS vectors, ST depression and/or T-wave inversion, and P-wave abnormalities, such as left atrial enlargement (7, 8). VF frequency was consistently lower in patients with an increased LV diameter (9). However, these ECG changes were neither sensitive nor specific for increased LV-D or LV-S detection. The EF serves as an indicator for cardiac contractility and a significant predictor of survival (10–13). Previous studies presented that LV-D increase was an independent risk factor of cardiovascular outcomes (14, 15), ventricular arrhythmia inducibility (16), and mortality (17, 18). By the investigation of 1,138 patients with HFrEF and sinus rhythm, Ito et al. proposed strong association between LV diameters and cardiovascular (CV) outcomes, which is independent of ECHO-EF (14). Moreover, in a combination with QRS duration, the LV-D could be applied to identify the patients at risk for tachyarrhythmias. Makaryus et al. revealed myocardial infarction with scar formation or cardiomyopathy with disordered ventricular excitation accounts for the ventricular arrhythmia and poor prognosis in patients with dilated LV-D (16). In patients with mitral regurgitation, the LV-S increase is independently associated with increased mortality even under medical management (19). All the results highlight the significance of EF, LV-D, and LV-S in patients with HF.

Artificial intelligence-based ECG (AI-ECG) has expanded to multiple applications and achieved human-level performance, effectively detecting cardiac diseases with large annotated ECG datasets, including echocardiogram predictions (20, 21), arrhythmia detection (22), dyskalemia and its cause (23–25), glycated hemoglobin (26), digoxin toxicity (27), aortic dissection (28), pneumothorax (29), and myocardial infarction (30–32). Importantly, previous studies revealed significant correlation and predictability between ECG-predicted EF (ECG-EF) and echocardiographic EF (ECHO-EF). This study not only revealed the diagnostic value of ECG on HF but also further identified a new subtype of HF, which has normal ECHO-EF but lower ECG-EF and a high risk of future LV dysfunction (20). Meanwhile, age estimated from ECG (ECG-age) is also a measure of cardiovascular health, and the difference between the ECG-age and the chronological age can be used as a marker of the risk of deaths even in different cohorts (33). The new concept of disease previvor was proposed as individuals who are healthy but have a markedly increased predisposition to develop the disease (34, 35).

However, the discrepancy between ECG-EF and ECHO-EF was not fully interpreted. ECHO-EF is evaluated regularly in echocardiography, similar to other cardiac structure measurements, such as LV-D, LV-S, interventricular, and posterior wall thickness. With the aid of AI-ECG, we hypothesized that AI-ECG predicting LV-D (ECG-LV-D) may provide additional information on CV outcomes in patients with initially normal ECHO-EF, who are recognized as low ECG-EF. Therefore, the aim of this study is to build a deep learning model (DLM) to predict LV-D and LV-S and verify the accuracy by echocardiography in two independent hospitals. Finally, we tried to apply ECG-LV-D in different clinical scenarios and acquire additional information on the prediction of future CV diseases.

## Materials and Methods

### Data Source and Population

**FIGURE 1:**
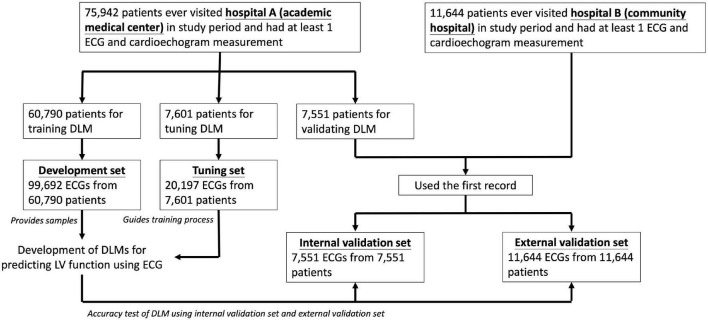
Development, tuning, internal validation, and external validation sets generation and the ECG labeling of echocardiogram. Schematic of the dataset creation and analysis strategy, which was devised to assure a robust and reliable dataset for training, validating, and testing of the network. Once a patient’s data were placed in one of the datasets, that individual’s data were used only in that set, avoiding “cross-contamination” among the training, validation, and test datasets. The details of the flowchart and how each of the datasets was used are described in “Materials and Methods” section.

This multicenter retrospective study was ethically approved by the institutional review board of Tri-Service General Hospital, Taipei, Taiwan (IRB NO. C202105049). The electronic medical records (EMRs) of our hospital included digital ECG signals, echocardiography images, hospital courses records, and future outcomes between 1 January 2010 and 31 September 2021. We identified patients who had at least one pair of 12-lead ECG and transthoracic echocardiography (TTE) records within 7 days. Subjects with inadequate ECG or echocardiographic information were excluded, such as noise interference, leads dislodge or dislocation, data loss of heart rate, EF, LV-D, or LV-S. The remaining ECGs were annotated by TTE information collected in this study. Finally, there were 75,942 patients in NeiHu General Hospital at NeiHu District (hospital A), an academic medical center in our hospital system, and 11,633 patients in Tingzhou Branch Hospital at Zhongzheng District (hospital B), a community hospital (Figure 1).

We divided ECGs into development, tuning, internal validation, and external validation sets by different dates and hospitals. For DLM training, there were 99,692 ECGs from 60,790 patients included in development set and 20,197 ECGs from 7,601 patients were included in tuning set. We only used the first records in the validation step for the patients with multiple ECG-TTE pairs, and the internal and external validation sets included 7,551 ECGs before 31 December 2015 in hospital A and 11,644 ECGs in hospital B. No repeated patients were recruited into more than one group.

### Observational Variables

The ECGs were acquired at a sampling rate of 500 Hz with a 10-s period using a Philips 12-lead ECG machine (PH080A, Philips Medical Systems, 3000 Minuteman Road Andover, MA 01810 United States). Comprehensive 2D ECG and quantitative data were recorded at the time of the acquisition in a Philips image system for all patients. The LV parameters included EF, LV-D, and LV-S, which were routinely acquired by experienced cardiologists or technicians using standardized methods. The EF was assessed using the Simpson method, M-mode, and the reported visually estimated EF. LV dimensions and wall thickness were measured by M mode under para-sternal long axis view and recorded by millimeter. The cut-off values of EF are 50 and 35% as mild and severe LV dysfunction, which are comparable criteria described in previous studies (20, 34, 36, 37). We selected LV-D and LV-S as they can be measured more easily and are more reproducible than other indices. Patients were divided into three groups according to LV-D at initial echocardiography: ≤ 56 mm (normal), 56 < LV-D ≤ 65 mm (mild increase), and > 65 mm (severe increase). The criteria for LV-S were ≤ 38 mm (normal), 38 < LV-S ≤ 45 mm (mild increase), and > 45 mm (severe increase). These diameters were determined according to the reference values for LV size from studies based on ethnic-appropriate population datasets (18, 38–43).

The demographic characteristics were obtained in our EMRs and disease history before the index date of ECG was collected using the corresponding code of International Classification of Disease, Ninth Revision and Tenth Revision (ICD-9 and ICD-10, respectively), as described previously (24, 26, 32, 44). The remaining echocardiographic parameters, such as interventricular septum (IVS) diameter, left ventricular posterior wall (LVPW) diameter, left atrium (LA) size, aortic root (AO) diameter, right ventricular (RV) diameter, pulmonary artery systolic pressure (PASP), and pericardial effusion (PE), were also collected in this study.

According to the promising ability of disease previvor identification by AI-ECG, we analyzed the correlation between ECG-LV-D increased and new-onset LV dysfunction, defined as ECHO-EF ≤ 35. Moreover, patients’ data were censored at the last known TTE examination to limit bias from incomplete records. In addition to LV dysfunction, we followed and analyzed other three CV outcomes, including CV mortality, new-onset acute myocardial infarction (AMI), and new-onset coronary artery disease (CAD). CV mortality included arrhythmia-related death, acute coronary syndrome-related death, stroke death, and HF-related death. These outcomes were censored at the patient’s last known hospital alive encounter without corresponding events to limit bias from incomplete records. The end of follow-up in this study was 30 September 2021 for all the above outcomes.

### The Implementation of the Deep Learning Model

The ECG-based EF, LV-D, and LV-S were, respectively, considered as function score and structure status of the heart, both estimated by DLMs. The ECG12Net architecture with 82 convolutional layers and an attention mechanism was used for estimation and the technology details, such as model architecture, data augmentation, and model visualization, were described previously (24). We used an oversampling process to adequately recognize extreme EF, LV-D, and LV-S values. The process was based on weights computed based on the prevalence of 20 equidistant intervals in the development set. The output of these DLMs was a continuous estimation value of actual EF, LV-D, and LV-S, which was called ECG-EF, ECG-LV-D, and ECG-LV-S, respectively.

### Statistical Analysis and Model Performance Assessment

Patient characteristics are presented as numbers of patients, population percentages, means, and standard deviations (SDs), with the significance level set as *p* < 0.05. We used scatter plots to describe the predicted value by ECG voltage-time traces compared with actual EF and left ventricular diameters (LV-D/LV-S). The accuracy of DLMs was evaluated by mean difference (Diff), Pearson’s correlation coefficients (*r*), and mean absolute errors (MAEs), calculated in both internal and external validation sets. The diagnostic value of DLMs was measured with the receiver operating characteristic (ROC) curve and the area under the curve (AUC). The tuning set was used to decide the operating point based on the maximum of Yunden’s index, which was calculated for the corresponding sensitivity, specificity, positive predictive value (PPV), and negative predictive value (NPV) in both validation sets. To identify the underlying subtype of patients with no correspondence between ECG-EF and ECHO-EF, the proportion of patients with larger ECHO/ECG-LV-D were explored in diverse ECHO/ECG-EF groups for the disease previvors of future LV dysfunction.

The relationship between traditional ECG features and AI-ECG-based ECG-LV-D was also analyzed. We showed the importance rank of different traditional ECG features, including 31 diagnostic pattern classes and 8 continuous ECG measurements based on an automatic Philips analysis system. These features were used to train an eXtreme gradient boosting (XGB) model to predict ECG-LV-D. To identify the most important ECG features in this analysis, the stepwise program was used and the *p*-value to enter and to remove were 0.05 and 0.15, respectively.

To investigate the long-term incidence of developing new-onset LV dysfunction, we plotted Kaplan–Meier curves of patients with an initially normal EF (EF > 50%), stratified by ECG-EF, left ventricle (end-diastole) diameter (LV-D), and ECG-LV-D. Multivariable Cox proportional hazard models were used to evaluate the predictive ability of ECG-EF, ECHO-LV-D, and ECG-LV-D adjusted by gender and age on all outcomes of interested, presenting in hazard ratios (*HR*s) and 95% conference intervals (95% *CI*s). We assessed the risk of adverse CV outcomes in patients with different ECG-EF/ECG-LV-D using a Cox proportional hazard model after adjusting by gender and age and demonstrated the risk matrixes of different outcomes with *HR*s and the concordance statistic (C-index), which were used to quantify their contributions. All the statistical analyses were conducted in R software, version 3.4.4.

## Results

The baseline characteristics of patients, including disease histories and echocardiographic data are presented in Table 1 for the development, tuning, internal validation, and external validation sets. In internal and external validation sets, 3,810 (50.5%) and 5,760 (49.5%) patients were men, and mean age was 63.4 and 65.7 years, respectively. According to disease history, there were 2,248 (29.8%) and 3,612 (31.0%) patients with diabetes mellitus (DM), 3,938 (52.2%) and 6,435 (55.3%) with hypertension (HTN), 3,125 (41.4%) and 5,176 (44.5%) with hyperlipidemia (HLP), 245 (3.2%) and 270 (2.4%) with AMI, 2,358 (31.2%) and 3,630 (31.2%) with CAD, and 957 (12.7%) and 1,484 (12.7%) with HF. The echocardiographic characteristics are similar between internal and external validation sets, such as EF (65.3%/65.5%), LV-D (47.4 mm/47.1 mm), and LV-S (29.8 mm/29.6 mm).

**TABLE 1:** Baseline characteristics.

	Development	Tuning	Internal validation	External validation
**Demography**				
Sex (male)	50,925 (53.6%)	10,600 (52.5%)	3,810 (50.5%)	5,760 (49.5%)
Age (years)	63.8 ± 17.4	68.0 ± 16.3	63.4 ± 16.6	65.7 ± 18.1
BMI (kg/m^2^)	24.6 ± 4.4	24.3 ± 4.4	24.5 ± 4.4	24.5 ± 4.3
**Disease history**				
DM	22,471 (23.6%)	7,211 (35.7%)	2,248 (29.8%)	3,612 (31.0%)
HTN	38,268 (40.3%)	11,778 (58.3%)	3,938 (52.2%)	6,435 (55.3%)
HLP	28,542 (30.0%)	9,088 (45.0%)	3,125 (41.4%)	5,176 (44.5%)
CKD	22,821 (24.0%)	8,820 (43.7%)	1,848 (24.5%)	2,896 (24.9%)
AMI	6,062 (6.4%)	2,099 (10.4%)	245 (3.2%)	279 (2.4%)
STK	13,055 (13.7%)	4,548 (22.5%)	1,274 (16.9%)	2,169 (18.6%)
CAD	26,382 (27.8%)	8,285 (41.0%)	2,358 (31.2%)	3,630 (31.2%)
HF	12,488 (13.1%)	4,777 (23.7%)	957 (12.7%)	1,484 (12.7%)
Afib	6,429 (6.8%)	2,570 (12.7%)	501 (6.6%)	754 (6.5%)
COPD	11,874 (12.5%)	4,372 (21.6%)	1,502 (19.9%)	2,758 (23.7%)
**Echocardiography data**				
EF (%)	63.6 ± 12.6	61.1 ± 14.2	65.3 ± 11.4	65.5 ± 10.8
LV-D (mm)	47.5 ± 7.1	47.9 ± 7.8	47.3 ± 7.1	47.1 ± 6.8
LV-S (mm)	30.3 ± 6.9	31.2 ± 7.8	29.8 ± 6.7	29.6 ± 6.3
IVS (mm)	11.2 ± 2.6	11.5 ± 2.6	11.2 ± 2.6	11.1 ± 2.6
LVPW (mm)	9.3 ± 1.7	9.5 ± 1.8	9.3 ± 1.7	9.1 ± 1.7
LA (mm)	38.4 ± 7.5	39.6 ± 8.0	38.6 ± 7.6	38.7 ± 7.3
AO (mm)	32.7 ± 4.4	33.1 ± 4.4	32.9 ± 4.5	32.8 ± 4.3
RV (mm)	23.7 ± 4.9	24.2 ± 5.1	24.1 ± 5.0	24.0 ± 5.0
PASP (mmHg)	33.3 ± 11.1	34.8 ± 12.4	32.2 ± 10.4	33.0 ± 10.7
PE (mm)	0.5 ± 2.1	0.6 ± 2.1	0.3 ± 1.8	0.4 ± 1.7

*BMI, body mass index; DM, diabetes mellitus; HTN, hypertension; HLP, hyperlipidemia; CKD, chronic kidney disease; AMI, acute myocardial infraction; STK, stroke; CAD, coronary artery disease; HF, heart failure; Afib, atrial fibrillation; COPD, chronic obstructive pulmonary disease; EF, ejection fraction; LV-D, left ventricle (end-diastole); LV-S, left ventricle (end-systole); IVS, Inter-ventricular septum; LVPW, left ventricular posterior wall; LA, left atrium; AO, aortic root; RV, right ventricle; PASP, pulmonary artery systolic pressure; PE, pericardial effusion.*

**FIGURE 2:**
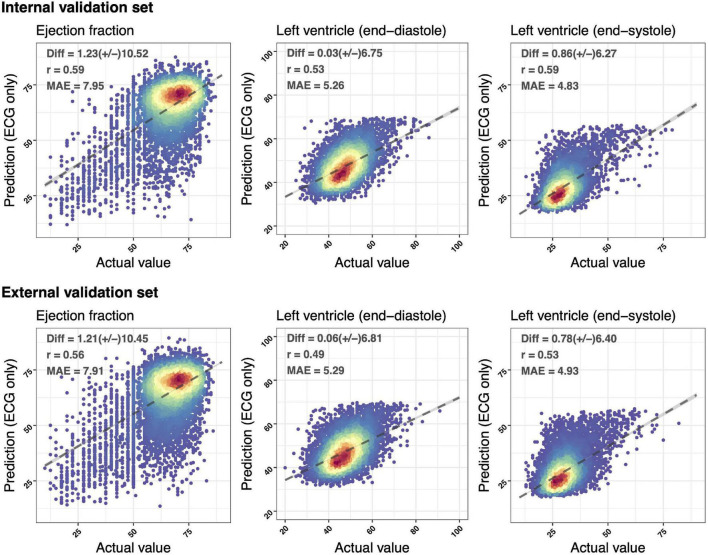
Scatter plots of predicted value *via* ECG voltage–time traces only compared with the actual ejection fraction (EF) and left ventricle (end-diastole/end-systole) (LV-D/LV-S). The *x*-axis indicates the actual value and the *y*-axis presents the ECG-predictions. Red points represent the highest density, followed by yellow, green light blue, and dark blue. We presented the mean difference (Diff), Pearson’s correlation coefficients (*r*), and mean absolute errors (MAEs) to demonstrate the accuracy of a deep learning model (DLM). The black lines with 95% conference intervals (*CI*s) are fitted *via* simple linear regression.

Figure 2 demonstrated the accuracy of DLMs with the scatter plots of ECG-based LV parameters compared to actual ones. The ECG-EF showed a high correlation with the Diff of 1.23 ± 10.52/1.21 ± 10.45, Pearson’s correlation coefficients (*r*) of 0.59/0.56, and MAEs of 7.95/7.91 in the internal/external validation set, respectively. Meanwhile, the similar correlation was observed in our analysis of ECG-LV-D and ECG-LV-S, with Diff of 0.03 ± 6.75/0.86 ± 6.27, *r* of 0.53/0.59, and MAE of 5.26/4.83 in the internal validation set, and Diff of 0.06 ± 6.81/0.78 ± 6.40, *r* of 0.49/0.53, and MAE of 5.29/4.93 in the external validation set.

**FIGURE 3:**
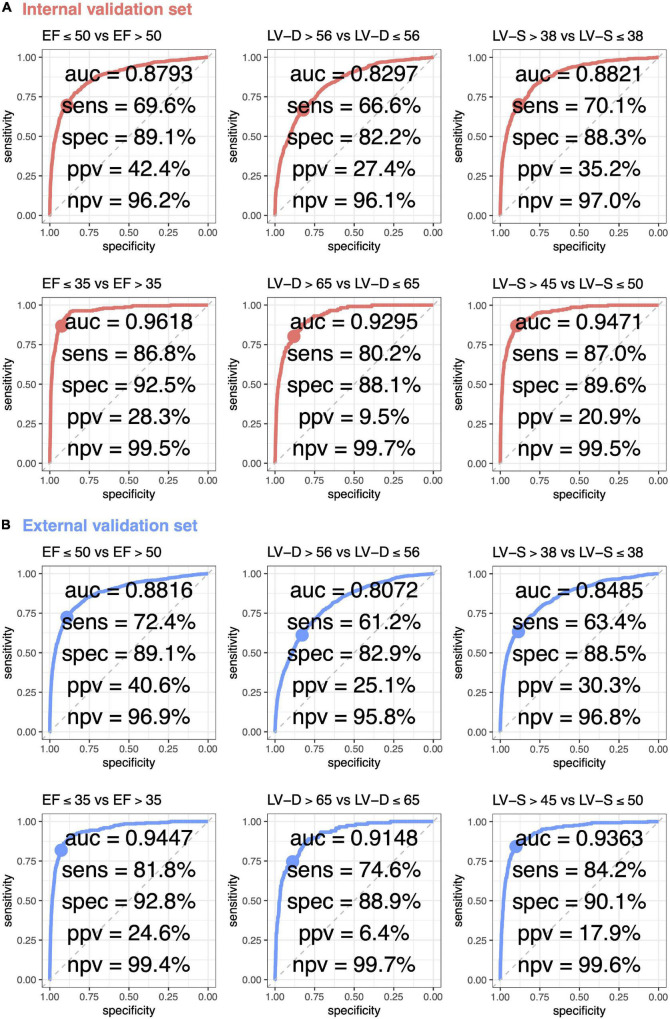
Receiver operating characteristic (ROC) curve analysis for mild to severe left ventricle abnormality from deep learning model based ECG voltage–time traces. The ROC curve (*x*-axis = specificity and *y*-axis = sensitivity) and area under ROC curve (AUC) were calculated using the internal validation set **(A)** and external validation set **(B)**. The operating point was selected based on the maximum of Yunden’s index in tuning set, which was used for calculating the corresponding sensitivities and specificities in two validation sets.

The ROC curve analysis was used to test the diagnostic value of AI-enabled ECG parameters (Figure 3). The AUCs of ECG-EF for mild/severe reduced EF in the internal validation set were 0.8793/0.9618, with a percentage of sensitivity of 69.6/86.8, specificity of 89.1/92.5, PPV of 42.4/28.3, and NPV of 96.2/99.5. Meanwhile, the AUCs of ECG-LV-D for detecting mild/severe increased ECHO-LV-D were 0.8297/0.9295 with the percentage of sensitivity of 66.6/80.2, specificity of 82.2/88.1, PPV of 27.4/9.5, and NPV of 96.1/99.7, and the AUCs of ECG-LV-S were 0.8821/0.9471 with the percentage of sensitivity of 70.1/87.0, specificity of 88.3/89.6, PPV of 35.2/20.9, and NPV of 97.0/99.5. The external validation analysis validated the generalization ability of DLMs in a heterogeneous population (AUC = 0.8816/0.9447 in ECG-EF, 0.8072/0.9148 in ECG-LV-D, and 0.8485/0.9363 in ECG-LV-S). These results revealed the possibility to detect abnormal EF/LV-D/LV-S *via* ECG accurately.

**FIGURE 4:**
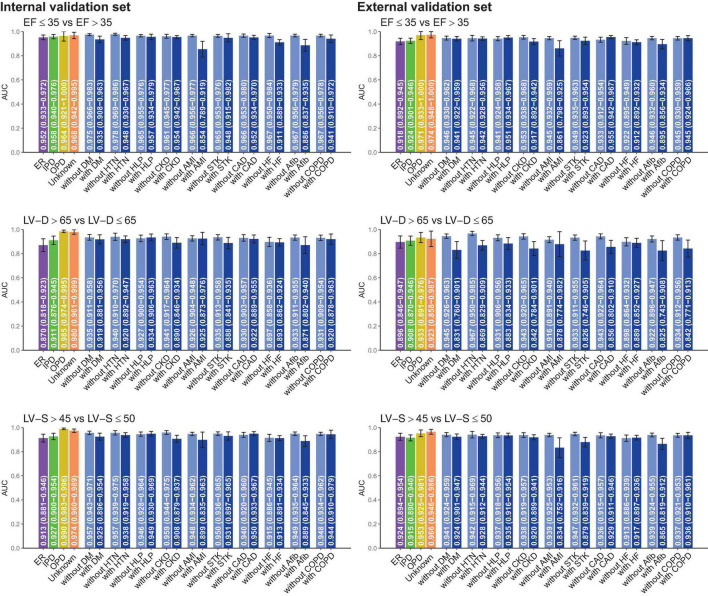
Stratified analysis for model performance for predicting electrocardiogram-based ejection fraction (ECG-EF), left ventricular end-diastolic diameter (LV-D), and left ventricular end-systolic diameter (LV-S). The analyses were stratified by the disease histories or data source. The AUC and 95% *CI*s were presented based on LV EFs and diameters. ER, Emergency room; IPD, inpatient department; OPD, outpatient department; DM, diabetes mellitus; HTN, hypertension; HLP, hyperlipidemia; CKD, chronic kidney disease; AMI, acute myocardial infarction; STK, stroke; CAD, coronary artery disease; HF, heart failure; Afib, atrial fibrillation; COPD, chronic obstructive pulmonary disease (COPD).

Subgroup analysis was stratified by the different clinical settings and comorbidities in Figure 4. DLM performed better in patients from the out-patient department (OPD) than those from the emergency room (ER) or the inpatient department (IPD). Compared to patients without comorbidities, ECG-EF, ECG-LV-D, and ECG-LV-S had lower AUC in patients with comorbidities, especially in patients with a history of AMI. These comorbidities may be potential confounding factors for new-onset LV dysfunction (45, 46). In other words, electrical abnormalities induced by comorbidities may cause ECG changes that interfere with the performance of our DLM.

**FIGURE 5:**
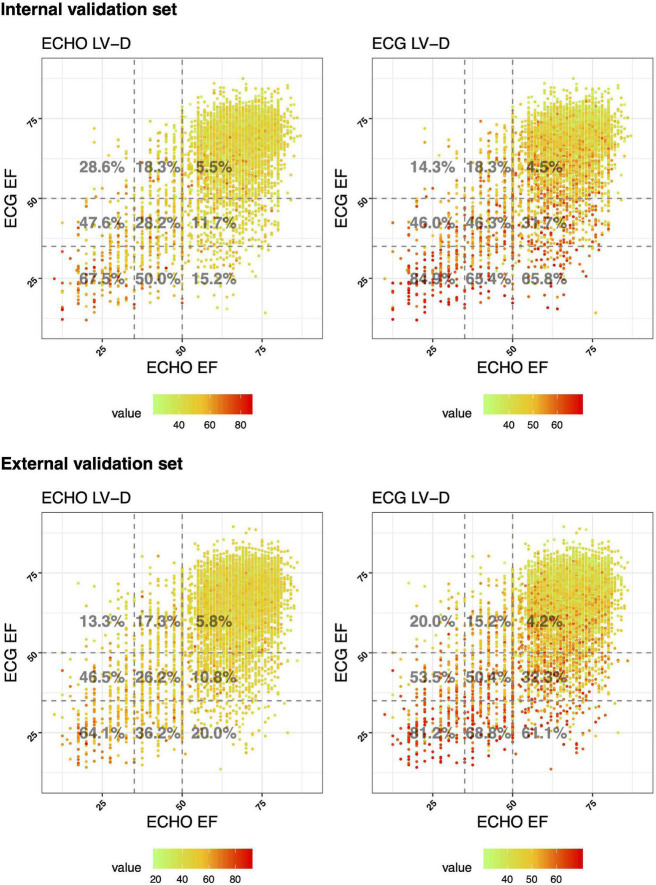
Scatter plots of predicted and actual EF correlated with LV-D. The *x*-axis indicates the actual EF and the *y*-axis presents the ECG-EF. Green to red points represent the small and large predicted and actual LV-D, respectively. The percentages were the proportion of people with an ECHO/ECG LV-D > 56 mm in each ECHO and ECG EF group.

In the previous study, we noticed patients with low ECG-EF and normal ECHO-EF had a higher incidence of future LV dysfunction. We hypothesized that the disease previvor was associated with obscure structural abnormalities, which could be detected by ECG-LV-D before actual LV dilation. Our DLM exhibited similar performance in predicting the size of LV-D and LV-S (Figure 3). Due to the similar clinical meaning of LV-S and LV-D in association with EF, we applied LV-D for further analysis. Figure 5 presents the scatter plots of predicted and actual EF correlated with LV-D. Initially, we applied ECHO-LV-D in the internal validation set but only 15.2% of patients with low ECG-EF and normal ECHO-EF were identified as the mild increase (>56 mm) in the internal validation set, however, the percentage increased to 65.8% in ECG-LV-D application group. In the external validation set, the percentage increased from 20.0 to 61.1% similarly. These results may reveal the importance of ECG-LV-D on previvors detection.

**FIGURE 6:**
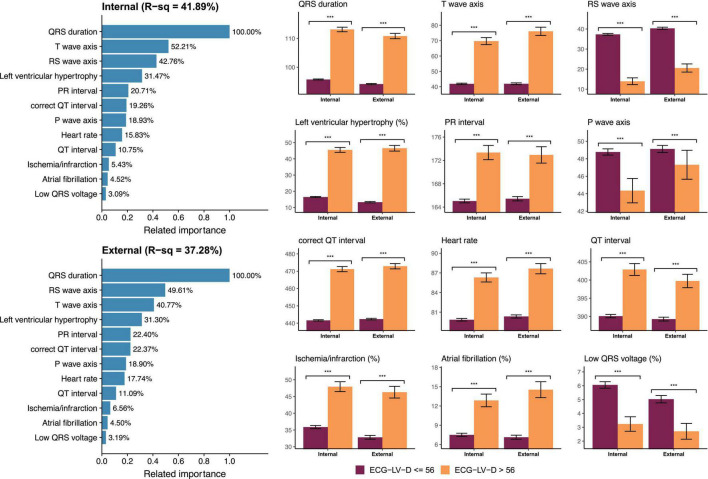
Relationship between most important ECG features and ECG estimated left ventricle (end-diastole) (ECG-LV-D). The related importance is based on the information gain of eXtreme gradient boosting (XGB) model, and the R-square (R-sq) is the coefficient of determination to use selected ECG features for predicting ECG-LV-D. The AI-ECG predictions were classified as ECG-normal (ECG-LV-D ≤ 56) and ECG-abnormal (ECG-LV-D > 56). The analyses are conducted both in internal and external validation sets (***p < 0.001).

Figure 6 demonstrated the relationship between known ECG features and ECG-LV-D. Our DLM identified those patients with increased ECG-LV-D were associated with the ECG features of ischemia/infarction, atrial fibrillation, tachycardia, left ventricular hypertrophy, widening QRS duration, prolonged PR interval, prolonged QT interval, augmented QRS amplitude, higher T-wave axis, lower RS wave axis, and lower P-wave axis compared to the ECG of normal patients. The explainable variation of known ECG features for DLM-based ECG-LV-D was 41.89 and 37.28% in the internal and external validation sets, respectively, which suggested that DLM could extract more than 50% additional information from raw ECGs.

**FIGURE 7:**
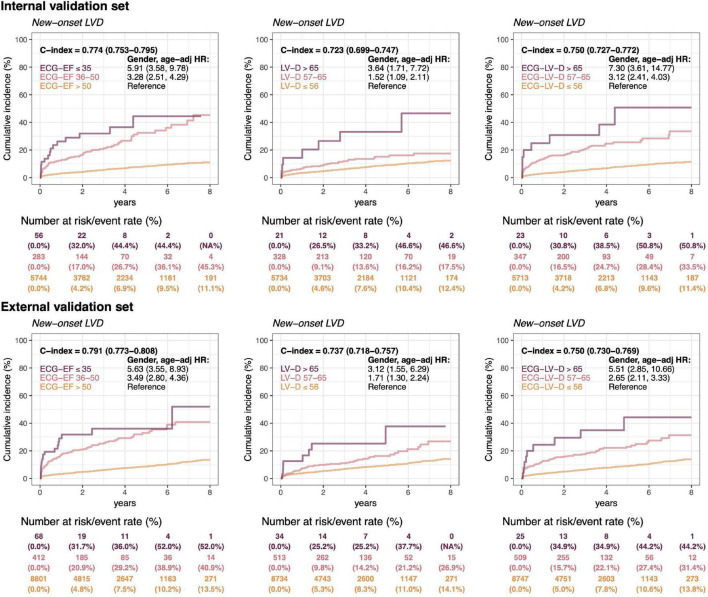
Long-term incidence of developing a new-onset left ventricular dysfunction (LVD, EF ≤ 35%) in patients with an initially normal EF (EF > 50%) stratified by ECG-EF, LV-D, and ECG-LV-D. The C-index is calculated based on the continuous value combined with sex and age. The analyses are conducted both in internal and external validation sets. The table shows the at-risk population and cumulative risk for the given time intervals in each risk stratification.

In Figure 7, a long-term incidence of developing a new-onset LV dysfunction in the patient with initially normal EF was presented. We stratified by ECG-EF, ECHO-LV-D, and ECG-LV-D and defined normal patient groups as reference. There were 6,083 patients and 9,281 patients at risk cases and the cumulative incidence rates in the low ECG-EF (false positive) group were percentages of 32.0/44.4/44.4 and 31.7/36.0/52.0 at 2/4/6 years in the internal and external validation sets, respectively, with corresponding significant gender-age adjusted *HR*s (95% *CI*) of 5.91 (3.58–9.78) and 5.63 (3.55–8.93). The C-index analyses also show the significant prognostic value on new onset LV dysfunction of 0.774 (95% *CI*: 0.753–0.7950) and 0.791 (95% *CI*: 0.773–0.808), which emphasized the importance of ECG-EF. In the analyses of ECHO-LV-D and ECG-LV-D, the significant gender-age adjusted *HR*s demonstrated the contributions on new-onset LV dysfunction in both validation sets. The *HR*s of severe/mild ECG-LV-D increase was 7.30 (95% *CI* 3.61–14.77)/3.12 (95% *CI* 2.41–4.03) in the internal validation set and 5.51 (95% *CI* 2.85–10.66)/2.65 (95% *CI* 2.11–3.33) in the external validation set. The C-indexes were higher in ECG-LV-D (0.750, 95% *CI* 0.727–0.772) than in ECHO-LV-D (0.723, 95% *CI* 0.699–0.747) in internal validation set, which was consistent in external validation set [0.750 (95% *CI*: 0.730–0.769) vs. 0.737 (95% *CI* 0.718–0.757)]. It suggested that ECG-LV-D may be a better differential indicator than ECHO-LV-D, which supplements the ECG-EF to identify patients at the risk of LV dysfunction in future.

**FIGURE 8:**
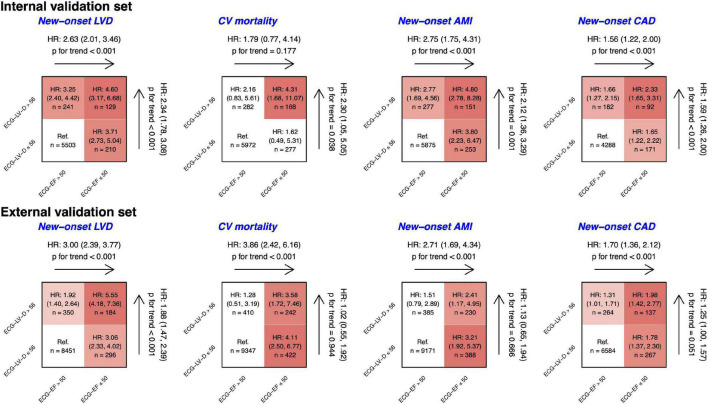
Risk matrixes of different predicted ejection fraction (ECG-EF) and left ventricle (end-diastole) (ECG-LV-D) groups on adverse events in patients with an initially normal EF (EF > 50%). The hazard ratios (*HR*s) are based on the Cox proportional hazard model adjusted by gender and age. The color gradient represents the risk of corresponding group, and the non-significant results are defined as white.

Figure 8 shows the risk matrixes of different ECG-EF and ECG-LV-D on adverse events in patients with normal ECHO-EF. The patients with increased ECG-LV-D were more susceptible to adverse CV outcomes. Combining ECG-EF and ECG-LV-D, the gender-age-adjusted *HR*s increased to 4.60 (95% *CI* 3.17–6.68), 4.31 (95% *CI* 1.68–11.07), 4.80 (95% *CI* 2.78–8.28), and 2.23 (95% *CI* 1.65–3.31) on new-onset LV dysfunction, CV mortality, new-onset AMI, and CAD, respectively. Moreover, the ECG-LV-D independently provided the ability of risk stratification on new-onset LV dysfunction (*HR* 2.34, 95% *CI* 1.78–3.08), CV mortality (*HR* 2.30, 95% *CI* 1.05–5.05), new-onset AMI (*HR* 2.12, 95% *CI* 1.36–3.29), and CAD (*HR* 1.59, 95% *CI* 1.26–2.00) in the internal validation set, and achieved similar trends with 1.88-fold-risk (95% *CI* 1.47–2.39) of new-onset LV dysfunction in the external validation set. In the consideration of confounding bias, we further adjusted more potential confounding factors, such as comorbidities. Our data indicated that the trend of results was similar with results adjusted by gender, age, and comorbidities (Supplementary Figure 1), which emphasized the importance and independency of ECG-EF and ECG-LV-D on early identification of HF risk.

## Discussion

In this study, we reported an AI-ECG DLM including more than 110,000 pairs of ECG and echocardiographic data and analyzed the longitudinal data, such as EF reduction, mortality, and adverse CV outcomes. Our DLM predicts ECG-EF accurately with the high AUCs of 0.9618/0.9447 for reduced EF detection (EF ≤ 35%) in the internal/external validation set, respectively. The high correlation between ECHO-EF and ECG-EF suggested the latter is a potential diagnostic tool. Severe/mild ECG-LV-D increase with the AUCs of 0.9295/0.8297 and 0.9148/0.8072 in internal/external validation set, which exhibited its valuable diagnostic power in patients with normal ECHO-EF. Moreover, we found a higher prevalence of ECG-LV-D increase in patients with low ECG-EF. Of these false positive patients, gender and age-adjusted *HR*s of future LV dysfunction were significantly high, suggesting that the DLM identified high-risk patients. Most importantly, the ECG-LV-D additionally contributes to predicting future LV dysfunction, which may provide the information of prognosis independently. The HRs of adverse CV outcomes increased significantly in patients identified as high ECG-LV-D and low ECG-EF compared with those with normal ECG-LV-D and ECG-EF. This is the first research to describe AI-enabled ECG-LV-D, which was demonstrated with high accuracy for the prediction of future LV dysfunction in patients with initially normal ECHO-EF.

Heart failure is an increasing problem affecting more than 30 million people globally. In these patients, asymptomatic LV dysfunction (ALVD, EF < 50%) patients are difficult to diagnose, who account for 7.9–23% of population (4, 5). Patients with ALVD were associated with the reduced quality of life, increased hospitalization, morbidity, and mortality (47, 48). Although current evidence highlights the significance of ALVD and emphasized the early intervention to these patients, there is no effective tool to screen patients with ALVD (49–53). In previous studies for LV dysfunction detection, Kwon et al. proved that the DLM outperformed other machine-learning methods (54). Even with different sex, age, and body mass index, Attia et al. and Cho et al. have demonstrated ECG-EF performance stability and robustness in internal and external validation sets (36, 55, 56). Our DLM exhibits excellent predictive performance in ECG-EF and ECG-LV-D. The concept of ECG-LV-D is proposed to expand the application of ECG-EF and tried to explain the discrepancy between ECG-EF and ECHO-EF. ECG-LV-D is thought to be a structural indicator with subtle electrical signal changes which provides critical information that helps to early identify those patients who are at risk to develop LV dysfunction. In combination with ECG-EF, the diagnostic power significantly enhanced, which could be applied for large-scale screening and for patients with asymptomatic HF to improve their CV outcomes.

There are several ECG changes in LV-D increase. In dilated cardiomyopathy (DCM), about 80% of patients had ECG abnormalities, including LV hypertrophy, left/right atrial enlargement, left/right bundle branch block, abnormal Q wave, atrial fibrillation, first-degree atrial-ventricular block, and T-wave inversion in inferior and anterolateral leads (57). Merlo et al. demonstrated that LV hypertrophy, increased heart rate, and anterior T-wave inversion predicted death or heart transplantation in patients with DCM and ECHO-EF < 50% (58). Previous studies proposed that delayed LV conduction with QRS prolongation (≥ 120 ms) was associated with restrictive LV filling, more advanced myocardial disease, worse LV function, poorer prognosis, and a higher all-cause mortality rate (59, 60). We found that our DLM was strongly correlated with prolonged QRS duration, which partly explained why the patients with high ECG-LV-D had a higher risk of LV dysfunction compared to patients with normal ECG-LV-D. Meanwhile, the possible mechanisms underlying the interference of DLM performance among patients with AMI could be myocardial scarring, which may affect electrical vectors, create regions of slowed conduction, and re-entrant circuits supporting sustained ventricular tachycardia (61, 62). Along with ECG-EF, the ECG-LV-D performed significantly better prediction capacity on new-onset LV dysfunction, CV mortality, new-onset AMI, and CAD compared to ECG-EF alone in the internal validation set. However, in the external validation set, in which the data from mild disease patients in community hospital, only the prediction of LV dysfunction could be significantly enhanced. Possible reasons underlying the inconsistency include different patient population and disease severities. Considering the better performance of our DLM in patients with less comorbidities from OPD than those from ER or IPD, our DLM could be more suitable for community screening than for hospitalized patients. Further large-scale studies are needed to confirm the combination effects of ECG-LV-D and ECG-EF.

The clinical application of AI-ECG is a worldwide tendency and developed rapidly. As the AI-ECG could predict the disease development in healthy individuals without abnormal imaging findings or symptoms, the concept of previvors was proposed recently. With apparent false positive AI-ECG findings, patients with a higher risk of many diseases, such as LV dysfunction (20), future atrial fibrillation (63), hyperkalemia (64), and elder heart age (44), could receive preventive interventions or medical surveillance early.

The importance and clinical significance of our ECG-LV-D should be emphasized. Both ECG-EF and ECG-LV-D are promising screening tools for patients who had a high risk of future LV dysfunction. The advantage of timely HF risk identification is evident to prevent adverse CV events and reduce medical costs. Moreover, from a large community-based study of sudden cardiac death (SCD), LV-D may contribute to the risk of SCD independent of the EF (41). The ECG-EF and ECG-LV-D models could be applied for risk stratification in patients with HF, especially those with stage A or B HF (65). Importantly, the wearable devices with ECG-EF and ECG-LV-D algorithms would provide timely conditions and beneficial effects for high-risk patients. Finally, considering that ECG is widely used and is a standardized examination in a rural or remote hospital, the AI-ECG could analyze and alert physician automatically and immediately among these areas. Further community-based studies of ECG-LV-D application are necessary to validate clinical benefits on HF patient care.

There are some limitations to this study. First, this study was a retrospective study. Although ECG/ECHO pairs were collected and the DLM was validated, the accuracy in different hospital settings and prospective studies are necessary to generalize the application of ECG-LV-D and promote treatment strategy. Second, the clinical impact of treatment is needed to verify. The actual benefit of ECG-LV-D import to clinical practice is not clear now. Investigation of clinical benefits including accidental HF detection, time reduction, prognosis management, and outcomes evaluation should be conducted. Third, the best application of AI-ECG is to screen asymptomatic patients with HF, but the relationship between abnormal ECG and HF symptoms was unclear. Future study should conduct a large-scale community screening to validate the benefit in asymptomatic patients with HF. Fourth, AI-ECG performed worse in patients with more comorbidities, especially in patients with a history of AMI. Interestingly, even after the adjustment of all the confounding factors, our models of ECG-EF and ECG-LV-D still provide significant predictive power for newly onset LV dysfunction. Finally, the DLM design is an uninterpretable set of methods, such as a black box, and full interpretability will be a focus of future work.

In conclusion, our AI-ECG DLM could identify patients with high ECG-LV-D and predict future LV dysfunction. ECG-LV-D serves as an independent risk factor of long-term CV outcomes in patients with normal ECHO-EF and low ECG-EF. The combination of ECG-EF and ECG-LV-D provides significantly synergistic diagnostic power to predict patients with future LV dysfunction. Although further studies are needed, our ECG-LV-D could be used as a screening tool for patients with normal EF but with high cardiovascular risk to initiate appropriate treatment in time.

## Data Availability Statement

The original contributions presented in the study are included in the article/Supplementary Material, further inquiries can be directed to the corresponding author/s.

## Author Contributions

CL, C-SL, and W-HF contributed to conception and design of the study. CL, C-HW, and C-CL organized the database. CL and C-SL performed the statistical analysis and wrote sections of the manuscript. H-YC and C-SL wrote the first draft of the manuscript. All authors contributed to manuscript revision, read, and approved the submitted version.

## Conflict of Interest

The authors declare that the research was conducted in the absence of any commercial or financial relationships that could be construed as a potential conflict of interest.

## Publisher’s Note

All claims expressed in this article are solely those of the authors and do not necessarily represent those of their affiliated organizations, or those of the publisher, the editors and the reviewers. Any product that may be evaluated in this article, or claim that may be made by its manufacturer, is not guaranteed or endorsed by the publisher.
