# Supplementary material for: Artificial Intelligence-Enabled Electrocardiogram Predicted Left Ventricle Diameter as an Independent Risk Factor of Long-Term Cardiovascular Outcome in Patients With Normal Ejection Fraction
Source: Front Med (Lausanne). 2022 Apr 11;9:870523. doi: 10.3389/fmed.2022.870523 (PMC9035739; doi:10.3389/fmed.2022.870523)
Supplement: Supplementary file 1 [file Image_1.pdf]

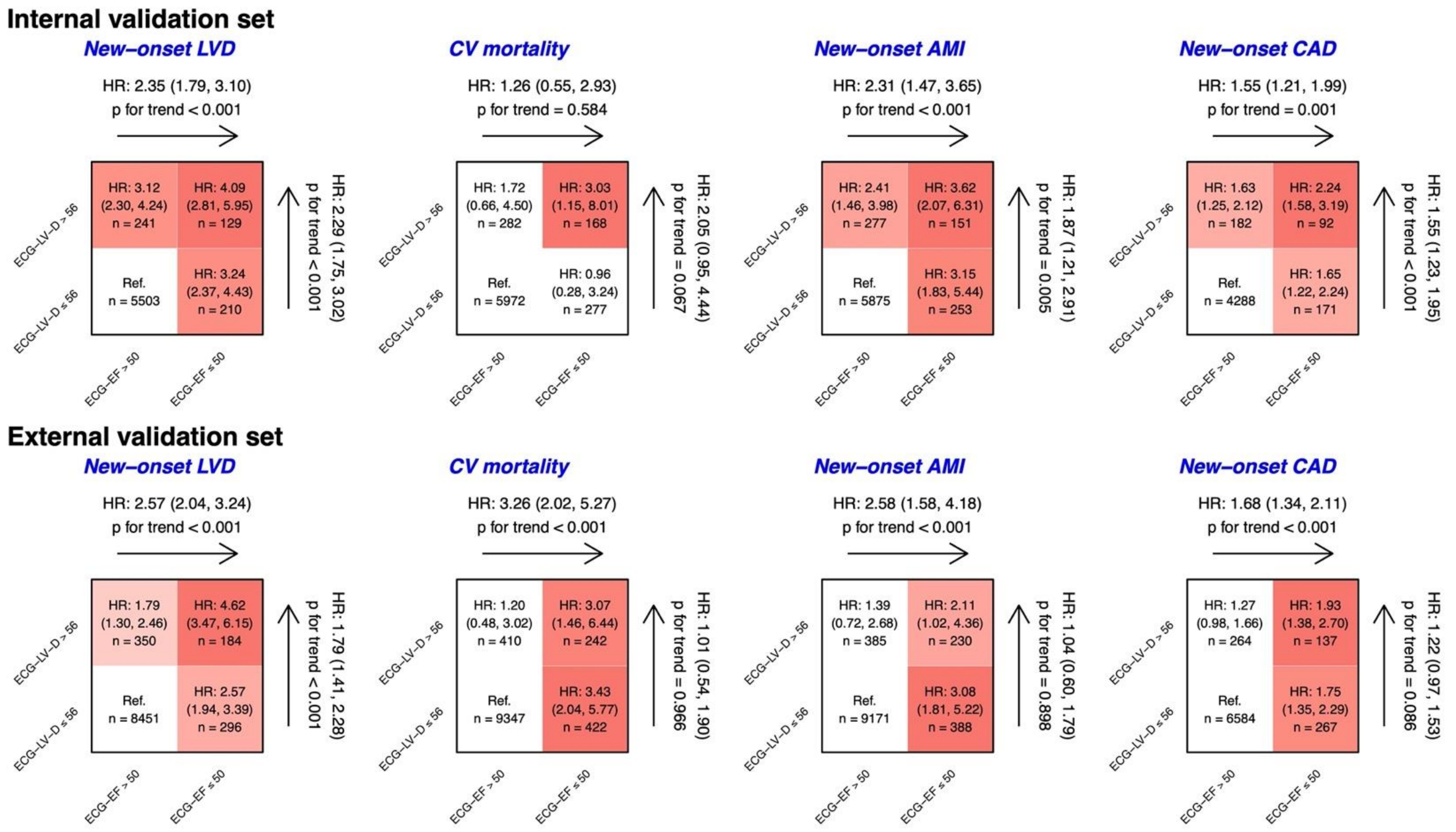

Supplementary Figure 1 | Risk matrixes of different predicted ejection fraction (ECG-EF) and left ventricle (end-diastole) (ECG-LV-D) groups on adverse events in patients with an initially normal EF (EF >50%). The hazard ratios (HRs) are based on Cox proportional hazard model adjusting by gender, age, and comorbidities. The color gradient represents the risk of corresponding group, and the non-significant results are defined as white.
